# The Circadian Clock Protein Timeless Regulates Phagocytosis of Bacteria in *Drosophila*

**DOI:** 10.1371/journal.ppat.1002445

**Published:** 2012-01-12

**Authors:** Elizabeth F. Stone, Ben O. Fulton, Janelle S. Ayres, Linh N. Pham, Junaid Ziauddin, Mimi M. Shirasu-Hiza

**Affiliations:** 1 Department of Neurobiology and Behavior, Columbia University Medical School, New York, New York, United States of America; 2 Department of Genetics and Development, Columbia University Medical School, New York, New York, United States of America; 3 Department of Microbiology and Immunology, Stanford University Medical School, Stanford, California, United States of America; Children's Hospital Boston, United States of America

## Abstract

Survival of bacterial infection is the result of complex host-pathogen interactions. An often-overlooked aspect of these interactions is the circadian state of the host. Previously, we demonstrated that *Drosophila* mutants lacking the circadian regulatory proteins Timeless (Tim) and Period (Per) are sensitive to infection by *S. pneumoniae*. Sensitivity to infection can be mediated either by changes in resistance (control of microbial load) or tolerance (endurance of the pathogenic effects of infection). Here we show that Tim regulates resistance against both *S. pneumoniae* and *S. marcescens*. We set out to characterize and identify the underlying mechanism of resistance that is circadian-regulated. Using *S. pneumoniae*, we found that resistance oscillates daily in adult wild-type flies and that these oscillations are absent in *Tim* mutants. *Drosophila* have at least three main resistance mechanisms to kill high levels of bacteria in their hemolymph: melanization, antimicrobial peptides, and phagocytosis. We found that melanization is not circadian-regulated. We further found that basal levels of AMP gene expression exhibit time-of-day oscillations but that these are Tim-independent; moreover, infection-induced AMP gene expression is not circadian-regulated. We then show that phagocytosis is circadian-regulated. Wild-type flies exhibit up-regulated phagocytic activity at night; *Tim* mutants have normal phagocytic activity during the day but lack this night-time peak. Tim appears to regulate an upstream event in phagocytosis, such as bacterial recognition or activation of phagocytic hemocytes. Interestingly, inhibition of phagocytosis in wild type flies results in survival kinetics similar to *Tim* mutants after infection with *S. pneumoniae*. Taken together, these results suggest that loss of circadian oscillation of a specific immune function (phagocytosis) can have significant effects on long-term survival of infection.

## Author Summary

Circadian rhythms, or daily oscillations in physiological functions, are nearly ubiquitous among animals. Though immune parameters are known to vary with time of day, circadian regulation of the immune system is not well understood. Studies of circadian biology and innate immunity are well established in the genetically tractable model organism, the fruit fly. We use this model to investigate the mechanisms underlying circadian-regulated immunity. We and others have found that flies infected at different times of day exhibit different mean survival times. There are three main components of fly immune defense against bacterial pathogens: antimicrobial peptide synthesis, reactive oxygen species generation, and phagocytosis, or the ingestion of microbes by host immune cells. Of these three, we found that only the phagocytic activity of host immune cells is circadian-regulated and peaks at night, during the rest phase. This night-time peak of phagocytosis correlates with the time of day of maximal resistance to infection by the bacteria *Streptococcus pneumoniae*. Mutants that lack circadian regulation also lack this night-time peak in phagocytosis. Inhibition of phagocytosis sensitized wild type flies to infection but did not further sensitize circadian mutants. These results suggest that this night-time peak in immune system function has significant effects on survival of infection.

## Introduction

Survival of bacterial infection is the result of complex host-pathogen interactions. An often-overlooked aspect of these interactions is the circadian state of the host. Most metazoans undergo daily, dynamic changes in their physiology that are regulated by well-characterized circadian machinery. At its core, the circadian clock is composed of four transcriptional regulators paired as two distinct heterodimers. In *Drosophila*, Period and Timeless form one heterodimer and Clock and Cycle form the other. These two heterodimers engage in an autoregulatory negative feedback loop that causes circadian oscillations in the expression levels of target genes [1]. Microarray analyses of different vertebrate tissues, such as heart, liver, or spleen-derived macrophages, have shown that approximately 5–10% of total gene expression in each tissue is circadian-regulated [2], [3]. These oscillations in transcription are thought to underlie circadian changes in the organism's physiology and behavior.

Reflecting the pervasiveness of circadian regulation, the *Drosophila* circadian mutants *Timeless* (*Tim*) and *Period* (*Per*) have pleiotropic phenotypes. They exhibit loss of circadian rhythms in locomotor activity, eclosion, male courtship, and oxidative stress response [4], [5], [6]. These circadian mutants are also sensitive to infection by at least two bacterial pathogens and resistant to infection by another [7], [8]. The circadian-regulated mechanisms underlying these changes in immunity were not known.

Two types of mechanisms can affect survival after infection: resistance and tolerance [9]. Resistance mechanisms control microbial growth, while tolerance mechanisms allow the host to endure the pathogenic effects of infection, including the host's response to infection. In *Drosophila*, known resistance mechanisms to control microbial growth in the hemolymph include antimicrobial peptide synthesis, reactive oxygen species generation, and phagocytosis by immune cells. Previously, microarray analysis comparing wild type and circadian mutants suggested that expression of immune signaling molecules that induce resistance mechanisms such as *Imd* (Immune deficient) may be circadian-regulated [10], [11]. Here we test the hypothesis that circadian mutants are sensitive to infection due to changes in mechanisms of resistance and investigate specific resistance mechanisms for circadian regulation.

It has been recently shown that in mice, many immune cell functions, such as cytokine secretion by macrophages and natural killer (NK) cells, undergo oscillatory time-of-day variation [3], [12]. Phagocytosis by immune cells such as macrophages and neutrophils has been reported to change with time of day, though it is not clear if this oscillation is diurnal (due to photoperiod) or circadian (regulated by circadian proteins) [13], [14], [15], [16]. Phagocytosis of photoreceptors by pigment cells in the vertebrate retina has also been shown to be circadian-regulated [17]. Thus we further hypothesized that phagocytosis of bacteria by immune cells in *Drosophila* is also circadian-regulated and that decreased phagocytic activity may significantly contribute to the sensitivity of circadian mutants to certain types of bacterial infection.

## Results/Discussion

### Circadian immune phenotypes are pathogen-specific

**Figure 1:**
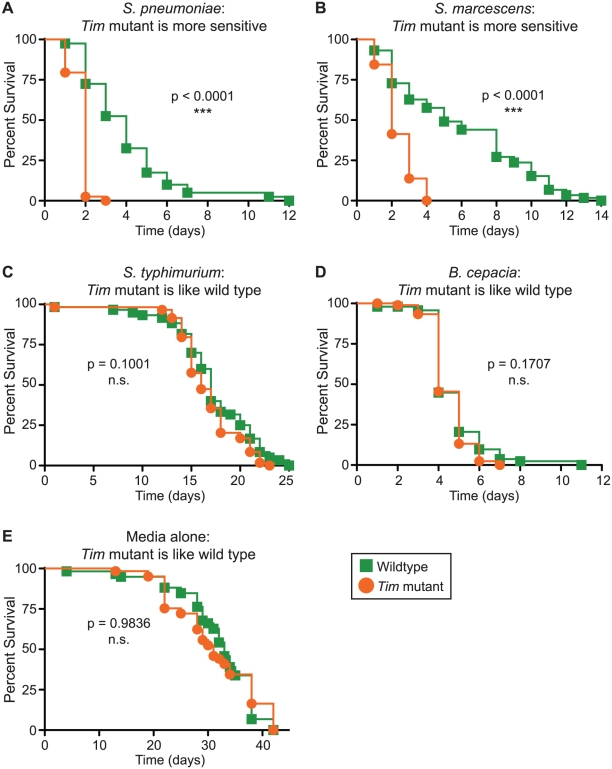
Tim activity has significant consequences for immunity. Shown here are Kaplan-Meier survival curves comparing *Tim* null mutants (orange circles) and wild-type control flies (green squares). *Tim* mutants die more quickly after infection with (A) *S. pneumoniae* (p<0.0001) or (B) *S. marcescens* (p<0.0001). *Tim* mutants die at the same rate as wild-type flies after infection with (C) *B. cepacia* (p = 0.1001), (D) *S. typhimurium* (p = 0.1707), or (E) medium alone (p = 0.9836). p-values were obtained by log-rank analysis.

The fly immune response to infection is highly complex and pathogen-specific. Previously, we showed that *Timeless (Tim)* null mutants die more quickly than wild-type flies when infected with *S. pneumoniae* [7]. Another group showed that *Tim* mutants die less quickly than wild-type flies when infected with *P. aeruginosa*, suggesting a complex immune phenotype that is pathogen-specific [18]. To further understand the immune phenotype of *Tim* mutants, we infected *Tim* mutants with three other pathogenic bacteria (*S. marcescens*, *B. cepacia*, and *S. typhimurium*). We compared *Tim* mutant survival time with wild-type flies and confirmed a complex immune phenotype (Figure 1, A–D). *Tim* mutants died more quickly than wild-type flies when infected with *S. marcescens* and died with wild-type kinetics when infected with *S. typhimurium* and *B. cepacia*. *Tim* mutants showed no difference in sensitivity to wounding alone. Thus, these data suggest that Tim activity has different consequences for immunity against different pathogens.

### Tim regulates resistance to specific bacterial infection

**Figure 2:**
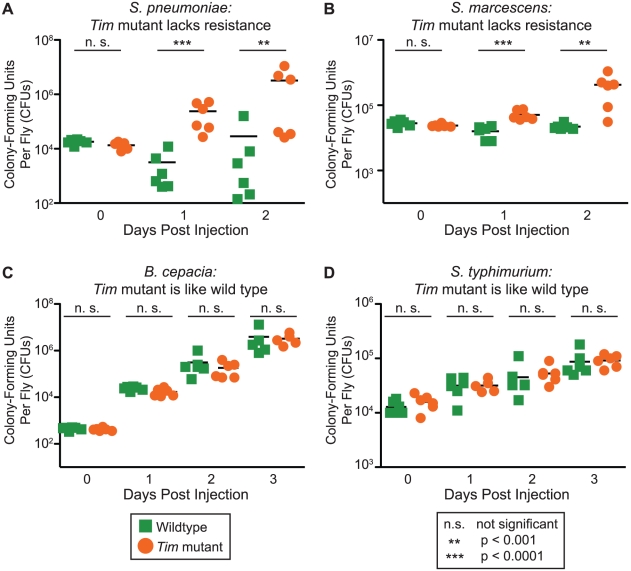
Tim regulates resistance to infection by *S. pneumoniae* and *S. marcescens*. Mutants lacking resistance, or bacterial killing, have shorter survival times and greater bacterial loads than wild-type flies. *Tim* null mutants lack resistance to (A) *S. pneumoniae* and (B) *S. marcescens*. *Tim* mutants have bacterial loads similar to wild-type flies when infected with (C) *B. cepacia* and (D) *S. typhimurium*. p-values were obtained by two-tailed, unpaired t-test; all experiments were performed with day-time injections.

We next asked if Tim regulates mechanisms of resistance or tolerance against specific bacterial pathogens [19]. We functionally distinguished between these two types of immune defense by comparing the bacterial loads of *Tim* mutant and wild-type flies after infection with four different bacterial pathogens (Figure 2, A–D). We found that loss of Tim protein causes a loss of resistance against two pathogens. *Tim* mutants die faster than wild-type flies when infected with *S. pneumoniae* and *S. marcescens* and have high bacterial loads. This suggests that *Tim* mutants are less able to control microbial growth and lack resistance to these two pathogens. *Tim* mutants had no effect on survival or bacterial growth during infection with *S. typhimurium* and *B. cepacia*.

These results demonstrate that Tim regulates resistance mechanisms for specific bacterial pathogens. Different physiologies will be important for defense against each type of pathogen; thus specific microbes can be used to probe different aspects of the immune system. Circadian proteins like Tim are known to regulate many different types of behavior and up to 5–10% of the transcriptome of different tissues. If specific physiologies that contribute to the defense against certain pathogens are circadian-regulated in *Drosophila*, then *Tim* mutants will present immune phenotypes when infected with those pathogens. Thus we can use our knowledge about the defenses required for different pathogens to identify those that are circadian-regulated.

### Light-induced degradation of Tim protein in wild-type flies causes sensitivity to S. pneumoniae

**Figure 3:**
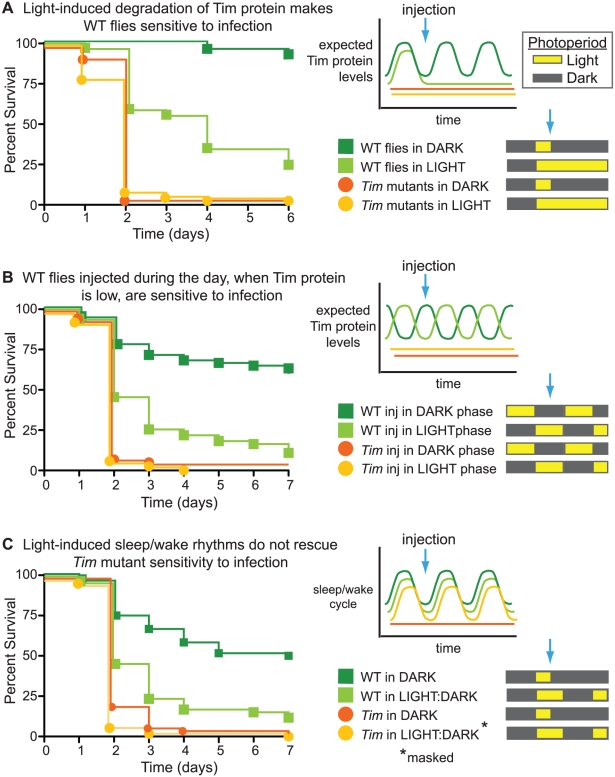
Resistance against *S. pneumonia* correlates with Tim activity. Shown here are Kaplan-Meier survival curves comparing wild-type flies and *Tim* mutants infected with *S. pneumoniae* and subjected to different light conditions as detailed in the schematic diagrams. (A) Light-induced degradation of Tim protein in wild-type flies results in increased sensitivity to *S. pneumoniae*. Infected wild-type flies incubated in constant light were more sensitive than wild-type flies incubated in darkness (p<0.0001). *Tim* mutants incubated in constant light died as rapidly as did *Tim* mutants incubated in darkness (p = 0.3404). (B) Wild-type flies were more sensitive to *S. pneumoniae* when infected at a time of day when Tim protein is low. Wild-type flies were less sensitive when infected 6 hours after lights off (DARK) than 6 hours after lights on, when Tim protein is minimally and maximally expressed, respectively (p<0.0001). *Tim* mutant sensitivity was not rescued by infection 6 hours after lights off (DARK) (p = 0.4134). (C) Light-induced sleep-wake rhythms (a startle response called “masking”) do not rescue *Tim* mutant sensitivity to infection. *Tim* mutant sensitivity was not rescued by incubation in cycling light:dark conditions compared to incubation in the dark. In fact, both *Tim* and wild-type flies were more sensitive to infection in cycling light:dark conditions than in the dark (p = 0.0125 and p<0.0001 respectively).

We chose to focus on *Tim* mutants and their dramatic defect in resistance against *S. pneumoniae*. We hypothesized that *Tim* mutants might lack resistance because of a developmental defect (such as malformation of immune tissues) or because of a direct effect of circadian disruption on immunity in the adult fly. To distinguish between these two possibilities, we disrupted Tim protein in genetically wild-type adult flies using constant light (Figure 3A). Blue light (∼380 to 475 nm) causes complete and rapid ubiquitin-mediated degradation of Tim protein [20]; normally, this allows the internal molecular clock to be synchronized with external light cues [4]. Flies reared in constant light exhibit complete loss of circadian rhythm [21]. Here we use constant exposure to light to induce a *Tim*-null phenocopy in adult wild-type flies, circumventing any developmental requirements for Tim protein. Wild-type flies were infected with *S. pneumoniae* and incubated either in darkness or constant light. We found that flies incubated after infection in constant light died faster than those incubated in darkness (p<0.0001). As a negative control, we treated *Tim* null mutants and found that they were not further sensitized by constant light, suggesting that *Tim* is epistatic to and therefore downstream of light treatment. These results are consistent with the hypothesis that Tim protein regulates resistance to *S. pneumoniae* in the adult fly and also demonstrate that environmental and genetic disruption of circadian regulation alters immunity.

### Wild-type flies are more sensitive to S. pneumoniae when infected at a time of day when Tim protein expression is low

If Tim protein positively regulates resistance in the adult fly and if Tim protein levels oscillate over the circadian day, we predicted that the resistance of wild-type flies should also oscillate over the circadian day. To test this, we infected two sets of flies, light-entrained in anti-phase with each other, at times that correspond to minimal and maximal Tim protein expression. Tim protein levels are lowest approximately 7 hours after lights turn on (Zeitgeber 07 or ZT07/day) and highest at approximately 7 hours after lights off (ZT19/night) [22]. Consistent with minimal and maximal *Tim* expression, wild-type flies infected during the day (ZT07) died significantly faster than those infected at night (ZT19) (Figure 3B). *Tim* null mutants showed no difference in survival time based on time of infection. These results suggest that increased *Tim* expression at night positively regulates a mechanism(s) of resistance required to fight infection by *S. pneumoniae*.

### Loss of resistance seen in Tim mutants cannot be rescued by a sleep/wake cycle induced by environmental light cues

“Masking” is a phenomenon in which flies lacking endogenous circadian rhythm exhibit circadian-like, light-stimulated locomotor activity and sleep-wake patterns due to external light cues (startle response) [23]. Thus *Tim* mutants in masking light/dark conditions exhibit a locomotor response to light stimulus even in the absence of Tim protein. We set out to ask if masking would restore the immune function of *Tim* mutants back to that of wild-type flies. To test this, we infected wild-type flies and *Tim* mutants with *S. pneumoniae* and incubated them either in the dark (relying on endogenous circadian rhythm) or in circadian light-dark conditions (masking) (Figure 3C). Masking conditions did not rescue the immune phenotype of *Tim* mutants infected with *S. pneumoniae*, suggesting that this phenotype is not rescued by light-stimulated locomotor activity but requires the normal expression of Tim protein.

### The melanization response is not circadian-regulated

We next examined the three main *Drosophila* immune responses for circadian regulation: melanization (generation of reactive oxygen species, or ROS), anti-microbial peptide (AMP) synthesis, and phagocytosis by hemocytes. We tested each immune response for time-of-day differences in wild type and *Tim* mutants and found evidence for circadian regulation of phagocytosis but not melanization or AMP gene induction. This is consistent with our survival data for *S. pneumonia*, as phagocytic hemocytes are crucial to control the growth of *S. pneumonia* in *Drosophila* [24].

**Figure 4:**
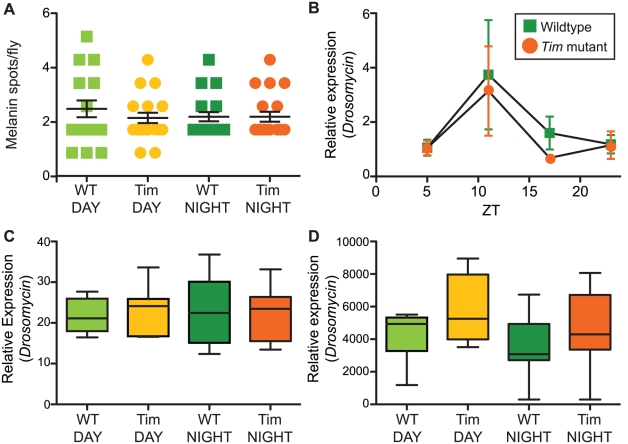
Tim protein does not regulate melanization or AMP gene expression. (A) Wild type and *Tim* mutants injected with *L. monocytogenes* at ZT07 (DAY) or ZT19 (NIGHT) have similar levels of melanization. p-values for pair-wise comparisons were not significant: WT (DAY) vs. Tim (DAY), p = 0.3551; WT (NIGHT) vs. Tim (NIGHT), p = 1.000; WT (DAY) vs. WT (NIGHT), p = 0.4031; Tim (DAY) vs. Tim (NIGHT), p = 0.8717. p-values were obtained by unpaired, two-tailed t-test; n = 18–20 flies per genotype per condition. (B) Basal (uninduced) levels of *Drosomycin* expression were increased at ZT11 relative to ZT05, ZT17, ZT23 in both wild type and *Tim* mutants, suggesting that this change in expression is not mediated by *Tim*. (C) Infection-induced levels of *Drosomycin* expression were not significantly different after injection of wild type and *Tim* mutants with *M. luteus* at ZT05 (DAY) or ZT 17 (NIGHT). Every pair-wise comparison resulted in p-values greater than 0.05 (not significant) by Mann-Whitney test. (D) Infection-induced levels of *Diptericin* expression were not significantly different after injection of wild type and *Tim* mutants with *E. coli* at ZT05 (DAY) or ZT17 (NIGHT). Every pair-wise comparison resulted in p-values greater than 0.05 (not significant) by Mann-Whitney test.

We first tested if the melanization response is circadian-regulated. Insects react to injury and some bacterial infections with an enzymatic cascade that generates toxic reactive oxygen species (ROS) and results in the deposition of melanin, visible through the cuticle as dark black spots [19]. If melanization is circadian-regulated, we would expect to see a difference in melanized spot formation between wild-type flies and *Tim* mutants as well as between wild-type flies at different times of day, but not between *Tim* mutants at different times of day. We assayed melanization in wild type and *Tim* mutants infected with *S. pneumoniae*, but did not observe a systemic melanization response, consistent with published reports [24], [25]. We then assayed the melanization response of flies infected with *L. monocytogenes*. Flies exhibit a strong melanization response after *L. monocytogenes* infection and require melanization enzymes for resistance against this pathogen [19]. We found no significant difference in melanized spot formation between wild-type flies and *Tim* mutants or between wild-type flies at different times of day (Figure 4A). These data suggest that melanization is not regulated by *Tim*.

### AMP gene expression induced by septic injury is not circadian-regulated

We next tested if antimicrobial peptide (AMP) gene expression is circadian-regulated. Expression of AMP genes in response to infection is the best characterized immune response of *Drosophila*; these small peptides secreted by the fat body are thought to control microbial growth by mechanisms such as disrupting bacterial membranes. Previously, others had found that expression of several immunity signaling genes controlling AMP gene expression such as *Rel* and *Imd* is circadian-regulated [8], [10], [11], [18]. Here we measured the basal (uninduced) and pathogen-induced expression of two representative AMP genes, *Drosomycin* (*Drs*) and *Diptericin (Dpt)*, which are widely used as reporters for induction of the *Toll* and *imd* signaling pathways, respectively [26].

We first compared the basal (uninduced) expression of *Drs* and *Dpt* in wild type and *Tim* mutants at four time points around the circadian cycle using qRT-PCR: ZT05, ZT11, ZT17, and ZT23. We found that both wild type and *Tim* mutants exhibited higher basal expression of the *Toll*-regulated AMP *Drs* at ZT11 than other time points (Figure 4B). This result suggests that basal expression of *Drs* varies with time of day but that this difference in expression is not mediated by Tim protein. This time-of-day difference in basal expression is statistically significant but very small relative to infection-induced expression levels (Figure 4 C, D). Basal expression of *Dpt* was not significantly different at any time of day in either wild type or *Tim* mutants (Figure S1A).

We next measured *Drs* and *Dpt* expression after *S. pneumoniae* infection in wild type and *Tim* mutants at ZT05 and ZT17, approximately when wild-type flies exhibit differences in survival and *Tim* mutants do not (see Figure 3). If AMP induction were circadian-regulated, we would expect to see a difference in AMP expression between wild-type flies at ZT05 and ZT17 and between wild type and *Tim* mutants at ZT17 but not between *Tim* mutants at ZT05 and ZT17. We found that *S. pneumoniae*-induced expression of *Drs* and *Dpt* was not significantly different at either time of day in either wild type or *Tim* mutants (Figure S1B, C).

*S. pneumoniae* is not typically used to induce AMP expression and did not cause the dramatic induction of AMP gene expression seen with other bacteria relative to media alone [27]. Thus we next examined circadian-regulation of AMP expression in response to two bacteria, *M. luteus* and *E. coli*, that have been most extensively used to probe AMP induction and more generally, to quantify signaling through the *Toll* pathway (activated by *M. luteus*) or the *Imd* pathway (activated by *E. coli*) [26]. We examined *Drs* and *Dpt* induction in wild-type flies and *Tim* mutants infected at either ZT07 or ZT19 with either *M. luteus* or *E. coli* and collected six hours later for qRT-PCR analysis. Again, we found that infection-induced expression levels of *Drs* and *Dpt* were not significantly different at these time points in wild type and *Tim* mutants (Figure 4C,D). Taken together, these data suggest that, for both *Drs* and *Dpt*, basal levels of expression and short-term expression induced by these bacteria (*S. pneumonia*, *M. luteus*, *E. coli*) are not circadian-regulated.

### Tim regulates phagocytosis of pathogens by immune cells

**Figure 5:**
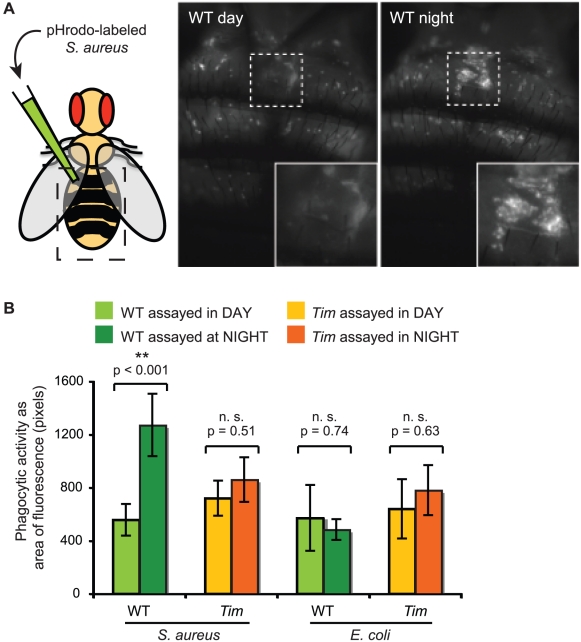
Tim protein regulates an early stage of phagocytosis of bacteria by hemocytes. (A) Wild-type flies in the night phase of the circadian cycle have more phagocytic activity than wild-type flies in the day phase. Shown here are images of the dorsal surface of representative flies after injection of dead *S. aureus* labeled with the fluorophore, pHrodo, which emits fluorescence in acidic environments such as the lysosomes. Hemocytes that have phagocytosed these bacteria become fluorescent. Dashed lines indicate the region enlarged in the inset. (B) Phagocytic activity for wild type and *Tim* mutant flies was quantified by measuring areas of fluorescence. Wild-type flies exhibited significant circadian differences in phagocytosis of *S. aureus* (p<0.001) but not *E. coli* (p = 0.74). This circadian difference is not present in *Tim* mutant flies with either *S. aureus* (p = 0.51) or *E. coli* (p = 0.63). p-values were obtained by unpaired, two-tailed t-test.

Finally, we tested for circadian regulation of the *Drosophila* immune response of phagocytosis—the physical engulfment and destruction of bacteria by specialized immune cells. Phagocytosis is typically assayed by measuring the internalization of fluorescently-labeled bacteria such as *S. aureus* or *E. coli*. Here, we measured phagocytic activity at different times of day in both wild-type flies and *Tim* mutants. If phagocytosis is regulated by Tim protein, we predict that phagocytic activity will be high at night and low during the day in wild-type flies but will not vary with time of day in *Tim* mutants. We assayed phagocytic activity in adult flies by injecting dead *S. aureus* labeled with pHrodo, a pH-sensitive rhodamine dye that fluoresces in acidic environments. When pHrodo-labeled bacteria are phagocytosed and processed into acidic lysosomes, the phagocytic cell emits red fluorescence and can be imaged through the dorsal surface of live, intact flies. We found that phagocytic activity was significantly higher at ZT19 (night) than ZT07 (day) in wild-type flies. Phagocytic activity did not vary with the circadian cycle in *Tim* mutants (Figure 5A, B). Thus, phagocytic activity oscillates with circadian rhythm *in vivo* in wild-type flies but not in *Tim* mutants, consistent with the hypothesis that Tim protein up-regulates phagocytosis of *S. aureus* at night. If phagocytosis is required to clear *S. aureus*, these data are consistent with previous studies demonstrating that wild-type flies exhibit higher rates of survival when infected with *S. aureus* at night than when infected during the day [18].

To test if circadian-regulated phagocytosis is bacteria-specific, we then assayed phagocytic activity with a different type of bacteria. We injected wild type and *Tim* mutants with pHrodo-labeled *E. coli* at ZT07 (day) and ZT19 (night) (Figure 5B). In contrast to injections of *S. aureus*, wild-type flies did not exhibit a circadian difference in phagocytosis of *E. coli*. *Tim* mutants also exhibited no circadian difference in phagocytosis of *E. coli*. These results suggest that *Tim* regulates bacteria-specific phagocytosis by immune cells.

Phagocytosis can be generally described as three steps: receptor-mediated substrate recognition and binding, particle engulfment, and phagosome maturation. Fluorescently-labeled *S. aureus* and *E. coli* have often been used in phagocytosis assays to determine whether specific phagocytic components discriminate between different types of bacteria. Many cellular steps of phagocytosis subsequent to substrate recognition do not appear to be bacteria-specific. For example, inhibition of β-COP, thought to be involved in phagosome maturation, leads to decreased phagocytosis of both *S. aureus* and *E. coli* [28]. In contrast, some molecular components responsible for phagocyte recognition of bacteria are bacteria-specific. For example, inhibition of the phagocytic receptor PGRP-LC leads to decreased binding and phagocytosis of *E. coli* but not *S. aureus* [28]. Another phagocytic receptor, PGRP-Sc1a, mediates phagocytosis of *S. aureus* but not *E. coli* [29]. Thus our finding that Tim up-regulates phagocytosis of *S. aureus* but not *E. coli* suggests that Tim regulates a bacteria-specific step of this process such as substrate recognition or binding.

### Bead inhibition of phagocytosis eliminates the survival difference between wild type and Tim mutants after infection with *S. pneumoniae*

**Figure 6:**
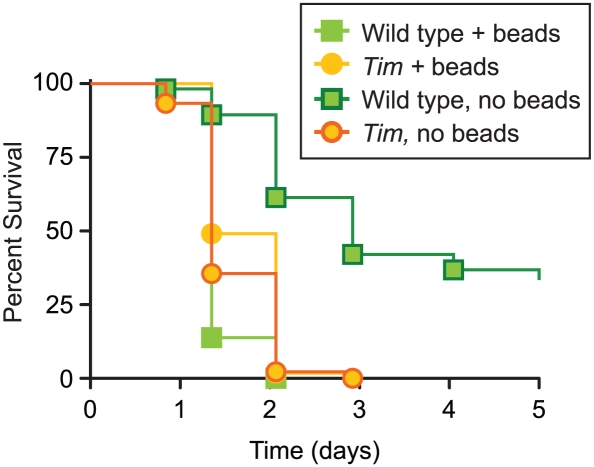
Bead inhibition of phagocytosis in wild type flies eliminates the difference in survival kinetics with Tim mutants after *S. pneumoniae* infection. Shown here are Kaplan-Meier survival curves comparing wild-type flies and *Tim* mutants infected with *S. pneumoniae* with and without pre-injection of beads to inhibit phagocytosis. Infected wild-type flies pre-injected with beads were more sensitive than wild-type flies without pre-injection (p<0.0001) and had similar survival kinetics as *Tim* mutants (p = 0.0502). Infected *Tim* mutants had identical survival kinetics with or without bead pre-injection (p = 0.1327). In this experiment infected, bead-injected wild-type flies were more sensitive than infected, bead-injected Tim mutants (p<0.0001); however, this result varied from experiment to experiment.

Because phagocytosis is crucial in defense against *S. pneumoniae* [24], these results suggest that differences in phagocytic activity might contribute to the difference in survival after *S. pneumoniae* infection between wild type and *Tim* mutants. Thus we tested if inhibition of phagocytic activity would decrease these survival differences. Phagocytosis can be inhibited *in vivo* by injection of polystyrene beads; phagocytes engulf these beads and are unable to phagocytose subsequent injections of fluorescently-labeled bacteria [30]. Thus we compared the survival kinetics of wild type and *Tim* mutants infected with *S. pneumoniae* with or without bead pre-injection (Figure 6). As described above, *Tim* mutants are highly sensitive to *S. pneumoniae* infection relative to wild type flies. Consistent with published results, we found that bead pre-injection increased sensitivity of wild type flies. Bead pre-injection of wild type flies decreased survival rate to those similar to *Tim* mutants, suggesting that inhibition of phagocytosis is sufficient to recapitulate *Tim* mutant sensitivity. Consistent with this, we also found that bead pre-injection did not increase the sensitivity of *Tim* mutants to *S. pneumoniae*, suggesting that phagocytosis is already impaired in these flies. Interestingly, in some experiments, bead-inhibited wild type flies appear to be more sensitive than bead-inhibited *Tim* mutants, suggesting that the presence of Tim protein in the absence of phagocytosis may have a negative effect on survival. Taken together, these results suggest that *Tim*-mediated phagocytosis plays an important role in survival of *S. pneumoniae* and that loss of *Tim* is equivalent to total inhibition of phagocytosis, though *Tim* mutants are still able to phagocytose. Inhibition of phagocytosis is thought to inhibit phagocytes' ability to signal the presence of infection; perhaps *Tim* also plays a role in these downstream signaling events. Importantly, our results do not rule out the possibility of multiple roles for Tim protein in immunity against bacterial infection, including *S. pneumoniae* infection.

Circadian regulation of immunity has long been reported anecdotally in humans and other animals. Here we show that Tim protein has complex, pathogen-specific effects on immunity, regulating resistance to infection in *Drosophila*. Resistance against *S. pneumoniae* is upregulated by *Tim* in the adult fly and is not simply a diurnal response (*i.e.,* activated by light/dark cycles even in the absence of circadian regulatory protein function). We also identified phagocytosis as a specific circadian-regulated immune mechanism. This is the first demonstration of a link between Tim protein and the cellular immune response *in vivo*. Our data suggests that circadian regulation affects phagocytic immune cells at an early stage of specific pathogen recognition and makes a significant contribution to survival of infection.

This work has implications for people whose occupations disrupt their circadian regulation such as night-shift workers, flight attendants, and hospital staff. Recently, it was shown that chronic jet lag (shifts in circadian rhythm) in mice did not cause loss of sleep or increased stress but did cause circadian dysregulation of the innate immune system and dramatic vulnerability to LPS-induced endotoxemic shock [31]. Furthermore, mice infected with *S. pneumoniae* during their rest phase died significantly less quickly than mice infected during their active phase [32]. In modern society, circadian dysregulation and exposure to bacterial infection at night are likely not uncommon. This work demonstrates that disruption of circadian oscillations in phagocytosis can have significant adverse effects on resistance against infection.

## Materials and Methods

### Fly and bacterial strains

The Canton S (CS) wild-type and CS *tim^01^* lines have been previously described [7]. Infections were performed with the following bacteria as described [33]: *Streptococcus pneumoniae* strain SP1, a streptomycin-resistant variant of D39 and gift from Elizabeth Joyce in Stan Falkow's laboratory at Stanford University [34]; *Listeria monocytogenes* strain 10403S, gift from Julie Theriot at Stanford University [35]; *Serratia marcescens* strain DB1140, gift from Man Wah Tan at Stanford University [36]; *Salmonella typhimurium* strain SL1344, gift from Falkow lab at Stanford University [37]; *Burkholderia cepacia* ATCC strain 2541; *Escheria coli* strain DH5-alpha; and *Micrococcus luteus* ATCC strain 4698. *S. pneumoniae* was frozen at OD_600_ 0.15 in 1 ml aliquots with 10% glycerol, pelleted upon thawing, rediluted three-fold in fresh BHI (Brain Heart Infusion media, Difco), and incubated (standing) for 1.5–2 hour at 37°C with 5% CO_2_ to OD_600_ 0.15 for injection into flies, which were incubated at 29°C. *L. monocytogenes* was grown in standing BHI, overnight at 37°C, and diluted to OD_600_ of 0.1 for injection into flies, which were incubated at 25°C. *S. marcescens* was grown in shaking BHI, overnight at 37°C, and diluted to OD_600_ ranging from 0.1 to 0.6 for injection into flies, which were incubated at 29°C. *S. typhimurium* was grown in standing LB, overnight at 37°C, and diluted to OD_600_ of 0.1 for injection into flies, which were incubated at 29°C. *B. cepacia* was grown in standing BHI, overnight at 29°C, and diluted to OD_600_ of 0.01 to 0.001 for injection into flies, which were incubated at 18° or 25°C. *E. coli* was grown in shaking LB, overnight at 37°C, and diluted to OD_600_ 0.1 for injection into flies, which were incubated at 25°C. *M. luteus* was grown in standing LB, overnight at 30°C, and diluted to OD_600_ 0.1 for injection into flies, which were incubated at 25°C.

### Injection

All experiments, including injections, were performed with male flies, 5–7 days post-eclosion. Flies were raised at 25°C, 55–65% humidity on yeasted molasses food in a 12h light/dark cycle. For injections, flies were anesthetized with CO_2_. Injections were carried out with a pulled glass capillary needle. A Picospritzer III (Parker-Hannifan) or custom-made Tritech microinjector (Tritech) was used to inject 50 nL of liquid into each fly. Volume was calibrated by measuring the diameter of the expelled drop under oil. Injections comparing ZT07 (day) and ZT19 (night) were conducted in a dark room using red safety lights as light sources.

### Survival assays

60–85 flies per genotype per condition were assayed for each survival curve and placed in 3 vials of dextrose food with approximately 20 flies each. In each experiment, 20–40 flies of each line were also injected with media as a wounding control. Death was recorded daily. Data was converted to Kaplan-Meier format using custom Excel-based software called Count the Dead. Survival curves are plotted as Kaplan-Meier plots and statistical significance is tested using log-rank analysis using GraphPad Prism software. All experiments were performed at least three times and yielded similar results.

### CFU determination

Following challenge with microbes, six individual flies were collected at each time point. These flies were homogenized, diluted serially and plated on appropriate media (tryptic soy blood agar for *S.pneumoniae*, LB for all others). Statistical significance was determined using non-parametric two-tailed t-tests. All experiments were performed at least three times and yielded similar results.

### Melanization assays

Flies were injected with *S. pneumoniae* (OD_600_ 0.1) or *L. monocytogenes* (OD_600_ 0.1) and incubated at 29°C. On day 2 of the *S. pneumoniae* infection or day 3 of the *L. monocytogenes* infection, flies were visually inspected for deposits of melanin resulting from the proteolytic cascade that generates reactive oxygen species. Injection with media typically causes a wound-induced melanization response, a small deposit of melanin at the site of injection within 24 hours. Infection with certain pathogenic bacteria such as *L. monocytogenes* or *S. typhimurium* also causes a disseminated melanization response, with large spots of melanin deposition observed elsewhere than the site of injection: under the cuticle or in deeper tissue on both dorsal and ventral sides of the abdomen, thorax, and head. Flies were quantified for both number and size of spots.

### Antimicrobial peptide gene expression analysis

Flies were injected with 50 nL of bacterial culture at OD_600_ 0.10 (*S. pneumoniae*, *M. luteus*, or *E. coli)*, or media alone (BHI, Difco). Following injection, flies were placed in vials containing molasses food and incubated at 25°C for five hours at the same light: dark cycle to which they had been entrained. Three groups of six flies were homogenized in Trizol and stored at −80°C until processed. RNA was isolated using a standard Trizol preparation and the samples treated with DNAse (Invitrogen). Fermentas RevertAid First Strand cDNA synthesis kit was used to produce the cDNA following the manufacturer's protocol. Quantitative RT-PCR was performed using a Stratagene Mx3000 qPCR machine, with Roche FastStart Universal SYBR Green Master (Rox) mix and the following primer sets: *Drosomycin*, *Diptericin*, *Rpl1* [38]. Total mRNA concentration was normalized using Rpl1 expression. Differences in the infection-induced gene expression were calculated by normalizing to basal gene expression in uninfected CS (day sample) taken at the same time point; p-values were obtained by Mann-Whitney test. The data shown in Figures 4 and S1 pool the results of three independent trials, each having three biological replicates of six flies in each sample for each condition.

### Phagocytosis assays

5–7 day old male flies were injected with 50 nL of 20 mg/ml pHrodo-labeled *S. aureus* or *E. coli* in water (Molecular Probes, cat# A10010 and P35361). The flies were allowed to phagocytose the particles for 30–60 min. The wings of the flies were removed and flies were pinned with a minutien pin onto a silicon pad. Fluorescence images were taken of the dorsal surface using epifluorescent illumination with a Leica MZ3 microscope fitted with an ORCA camera (Hamamatsu). Images were captured with Openlab (Improvision) software. The exposure was set such that the brightest images had a very small number of saturated pixels. The experiment was repeated three times with 6–10 flies for each treatment.

### Bead inhibition of phagocytosis

Fluorescent 0.2 um polystyrene beads (Molecular Probes, cat# F13080) were injected into hemolymph to inhibit phagocytosis as previously described [30]. Briefly, 200 ul of beads in solution were washed three times in sterile water and resuspended in 20 ul final volume. 5–7 day old male flies were injected with 50 nL of bead solution. To confirm that phagocytosis was inhibited with this protocol, the *in vivo* phagocytosis assay was performed as described above. Phagocytosis was completely inhibited within 2 days of bead injection. Flies were then injected with *S. pneumoniae* at OD_600_ ranging from 0.05–0.15 (see Survival Assays, above).

### Accession numbers

Accession numbers are listed below for the following genes, which were examined in this study. Accession numbers were obtained from www.uniprot.org: Timeless (P49021); Drosomycin (P41964); Diptericin (P24492)

## Supporting Information

Figure S1 **Tim protein does not regulate AMP gene expression.** (A) Basal (uninduced) levels of *Diptericin* expression were very low and not significantly different at different times of day in wild type and *Tim* mutants. Every pair-wise combination resulted in p-value greater than 0.05 (not significant) by Mann-Whitney test. (B) *Drosomycin* expression levels induced by injection of *S. pneumoniae* or media (BHI) are not circadian-regulated. Wild type and *Tim* mutants were injected at ZT05 (DAY) or ZT 17 (NIGHT). Pair-wise comparisons of flies injected with *S. pneumoniae* resulted in p-values greater than 0.05 (not significant) by Mann-Whitney test. *Tim* mutants injected with media during the day exhibited significantly less *Drosomycin* expression than wild-type flies injected with media during the day or night or *Tim* mutants injected at night; all other pair-wise comparisons of flies injected with media did not show significant differences by Mann-Whitney test. *S. pneumoniae*-infected flies exhibited higher levels of *Drosomycin* expression than BHI-injected controls; p<0.01 by Mann-Whitney test. (C) Infection-induced levels of *Diptericin* expression were not significantly different after injection of wild type and *Tim* mutants with *E. coli* at ZT05 (DAY) or ZT17 (NIGHT). Pair-wise comparisons within the set of flies injected with *S. pneumoniae* resulted in p-values greater than 0.05 (not significant) by Mann-Whitney test. *S. pneumoniae*-infected and BHI-injected flies did not exhibit significant differences in *Diptericin* expression; p>0.05 by Mann-Whitney test.
(EPS)
